# Radioiodinated Capsids Facilitate *In Vivo* Non-Invasive Tracking of Adeno-Associated Gene Transfer Vectors

**DOI:** 10.1038/srep39594

**Published:** 2017-01-06

**Authors:** P. Kothari, B. P. De, B. He, A. Chen, M. J. Chiuchiolo, D. Kim, A. Nikolopoulou, A. Amor-Coarasa, J. P. Dyke, H. U. Voss, S. M. Kaminsky, C. P. Foley, S. Vallabhajosula, B. Hu, S. G. DiMagno, D. Sondhi, R. G. Crystal, J. W. Babich, D. Ballon

**Affiliations:** 1 Citigroup Biomedical Imaging Center, Department of Radiology, Weill Cornell Medical College, New York, New York, USA; 2 Department of Genetic Medicine, Weill Cornell Medical College, New York, New York, USA; 3 Department of Medicinal Chemistry and Pharmacognosy, University of Illinois at Chicago, Chicago, Illinois, USA.

## Abstract

Viral vector mediated gene therapy has become commonplace in clinical trials for a wide range of inherited disorders. Successful gene transfer depends on a number of factors, of which tissue tropism is among the most important. To date, definitive mapping of the spatial and temporal distribution of viral vectors *in vivo* has generally required postmortem examination of tissue. Here we present two methods for radiolabeling adeno-associated virus (AAV), one of the most commonly used viral vectors for gene therapy trials, and demonstrate their potential usefulness in the development of surrogate markers for vector delivery during the first week after administration. Specifically, we labeled adeno-associated virus serotype 10 expressing the coding sequences for the CLN2 gene implicated in late infantile neuronal ceroid lipofuscinosis with iodine-124. Using direct (Iodogen) and indirect (modified Bolton-Hunter) methods, we observed the vector in the murine brain for up to one week using positron emission tomography. Capsid radioiodination of viral vectors enables non-invasive, whole body, *in vivo* evaluation of spatial and temporal vector distribution that should inform methods for efficacious gene therapy over a broad range of applications.

Gene therapy is a burgeoning field with wide-ranging applications that include cancer, cardiovascular, neurological, infectious, and chronic inflammatory diseases^1^. For *in vivo* gene therapy, where the gene is administered directly to the recipient, the gene is typically placed in a genetically modified virus which serves as the vehicle for efficient binding and entry to the target cells, and delivers the therapeutic gene to the nucleus where it functions to express the desired protein. Viral vector mediated gene administration has been an especially promising area of research, with the best candidate viruses possessing minimal pathogenic and immunogenic responses^2^. Adeno-associated virus (AAV) satisfies these criteria^3^,^4^,^5^,^6^,^7^,^8^,^9^,^10^,^11^,^12^,^13^,^14^,^15^,^16^,^17^,^18^. AAV is a small (23 nm) diameter virus with an icosahedral capsid, with an available genome payload of approximately 4.8 kilobases. This is sufficient for many applications, and there have been over 135 clinical trials using AAV reported thus far^1^.

One of the challenges of gene therapy administration is localization of the gene product to the target tissue/organ, and achievement of the appropriate viral concentration and spatial distribution therein. The gold standard for determining the spatial distribution of gene product in tissue is immunohistochemical staining, which requires either post-mortem access to tissues or biopsy. To meet this challenge, there has been great interest over the last fifteen years in various labeling techniques that render transfected tissue amenable to some form of *in vivo* imaging, whether it be through the use of optical bioluminescence or fluorescence, positron emission tomography (PET), single photon emission tomography (SPECT), or magnetic resonance (MRI)^19^.

Each imaging method has advantages and disadvantages. The optical techniques offer sub-micron spatial resolution and are inexpensive to perform but suffer from a very short penetration depth in tissue due to the absorption properties of optical photons. The physics of PET and SPECT limit the spatial resolution to a few cubic millimeters, but concentrations of imaging agents in the picomolar range can be detected and quantified. MRI offers practical spatial resolution down to about 50 microns, but concentrations of metabolites in the millimolar range are generally required for detection.

The focus of this study was to develop a direct imaging strategy that could be used to track the spatial and temporal distribution of the actual gene transfer vector independent of the transgene. To accomplish this, we used as a model the adeno-associated virus (AAV) vector AAVrh.10CLN2 that is currently being tested as gene therapy for late infantile neuronal ceroid lipofuscinosis (LINCL, CLN2 disease, Batten disease), a uniformly fatal inherited pediatric lysosomal storage disorder (ClinicalTrials.gov identifier NCT01161576)^20^,^21^,^22^. Mutations in the CLN2 gene disrupt production of tripeptidyl peptidase 1 (TPP-1), an essential lysosomal enzyme. A lack of adequate levels of TPP-1 results in a host of neurological problems in humans that ultimately results in death by the age of 10–12 years. We labeled the capsid of AAV serotype 10 with iodine-124 (I-124), which is a positron emitting radionuclide with a physical half-life of 4.18 days^23^. The chemistry of iodine is very favorable for covalent labeling of tyrosine residues that are present on the AAV capsid, and I-124 is an isotope of iodine that is readily available from medical cyclotrons.

In this paper we present two radiochemical methods for labeling the AAV capsid. The choice of isotope is of course independent of the chemistry, and I-124 could readily be replaced with longer-lived isotopes of iodine if desired. The selection of I-124 was motivated in part by the superior sensitivity of PET relative to SPECT and its translational potential to studies involving human subjects^24^. In the first method, we used the iodination reagent Iodogen, which facilitates direct iodination of the viral capsid^25^,^26^. This technique has been applied to many biomolecules but requires their exposure to oxidizing conditions. The second method made use of a modified Bolton-Hunter reagent. This technique involves the iodination of an acylating agent prior to its reaction with the viral capsid. It avoids exposing virus particles to an oxidant and so is potentially less damaging to the virions than Iodogen^27^,^28^.

Our goal is to present the chemistry necessary for radiolabeling in sufficient detail so that the techniques can be readily duplicated by experienced radiochemists, and also to examine the basic *in vivo* behavior of the radiolabeled product, including the duration of visible radioactivity via PET and the effect of the methods on the infectivity of AAVrh.10CLN2. For this purpose, the vector was administered via intraparenchymal injection to the striatum of wild type mice.

## Results

**Figure 1:**
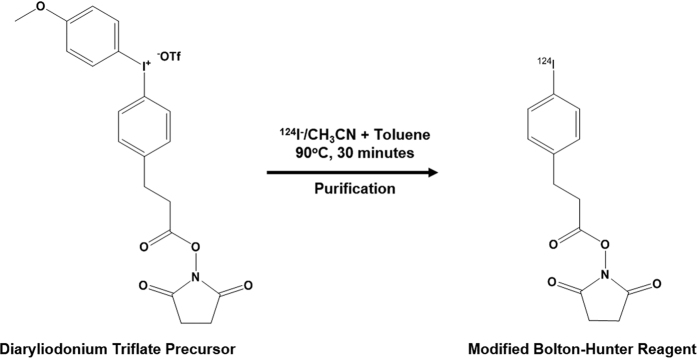
Modified Bolton-Hunter reagent labeling. The reaction of diaryliodonium trifluoromethane-sulfonate salt with [^124^I] NaI in acetonitrile/toluene solvent matrix produced an I-124 labeled modified Bolton-Hunter intermediate.

**Figure 2:**
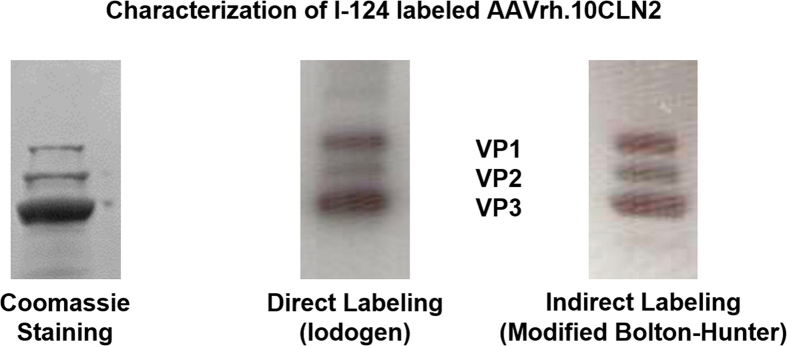
Gel electrophoresis assay to ascertain the identity of the labeled virus particle. Samples were heated to 80 °C for 3 minutes causing capsid disintegration, resulting in three bands corresponding to VP1, VP2, and VP3 capsid protein constituents. The purification process removed unreacted free iodide as well as aggregates formed during radiolabeling.

For the purpose of labeling the AAVrh.10 capsid with I-124, we tested two distinct radioiodination methods to determine the potential advantages of each. In first method, Iodogen [1,3,4,6-tetrachloro-3α,6α-diphenyl glycoluril] was used to oxidize iodide atoms to a positively charged species which covalently labels tyrosine residues on the capsid. The use of an oxidation step may adversely affect the biological activity of the substrate molecule, so we attempted to avoid direct exposure of the capsid to Iodogen by incubating separately the I-124 sodium iodide solution with Iodogen and then transferring the activated I-124 to a separate plastic tube containing the virions for labeling. The purification of the reaction mixture was achieved by passage through a cartridge containing a strong ion exchanger followed by filtration through a 100 K size exclusion filter, where radioiodinated AAVrh.10CLN2 was retained on the filter. Recovery of I-124AAVrh.10CLN2 was achieved after repeated washes with PBS. As an alternative, we tested a modified Bolton-Hunter method, which involved direct nucleophilic iodination of a diaryliodonium salt precursor (4-(3-N-hydroxysuccinimidylpropionyl)phenyl)(4-methoxyphenyl)iodonium triflate to form I-124–2,5-dioxopyrrolidin-1-yl 3-(4-iodophenyl)propanoate, isolating it from free I-124 (Fig. 1), and then reacting the radiolabeled acylating agent with the lysine residues of the capsid. The purification procedure was identical to that described above (Fig. 2).

**Figure 3:**
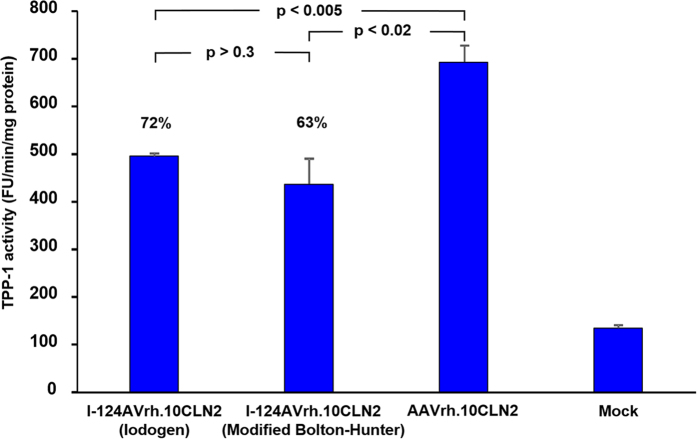
Activity of I-124AAVrh.10CLN2 vector *in vitro*. 293ORF6 cells in a 12-well plate (4 × 10^5^) were infected with I-124AAVrh.10CLN2 (10,000 genome copies/cell). At 72 hours post infection cells were harvested and a cell lysate was prepared (200 μl). Kinetics of TPP-1 activity were assessed with 20 μl every 10 min for 60 min using a Cytofluor 4000TC plate reader. The plate was read from the bottom using 360/20 nm excitation and 460/25 emission filters. The gain was set at 70. Uninfected cell lysate (Mock) served as the control. TPP-1 activity is expressed as mean fluorescence unit/min/mg protein ± standard error.

**Figure 4:**
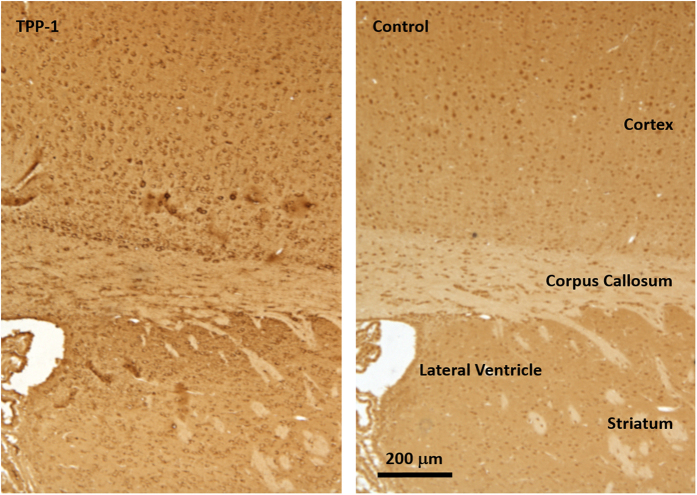
Immunohistochemical assessment of human TPP-1 expressed by I-124AAVrh.10CLN2 using the Iodogen method. Images are sequential coronal sections 5 μm apart and obtained on sacrifice, 4.5 weeks after vector administration. TPP-1 staining is observed in the cortex and striatum with halo-like patterns of cell bodies (left) as compared to background staining of the neighboring section using an irrelevant IgG (right).

Tests of labeling efficiency for the Iodogen method yielded up to an average of 13.36 I-124 atoms per virus particle while for the modified Bolton-Hunter technique we achieved up to 3.17 atoms per virus particle. It was therefore assumed that most virions were radiolabeled, so that an assay of infectivity would reflect the true effects of chemical and radiochemical manipulations. We tested the infectivity of I-124AAVrh.10CLN2 by measuring the ability of the vector to express TPP-1 after infection of 293ORF6 cells. The infectivity assay for Iodogen yielded TPP-1 activity that was 72% of the value observed for unlabeled virus (p < 0.005). For the modified Bolton-Hunter method the value was slightly lower at 63% (p < 0.02, Fig. 3), but the difference between Iodogen and modified Bolton-Hunter methods was not statistically significant (p > 0.3). In addition to the *ex vivo* tests using 293ORF6 cells, we were also interested in the *in vivo* performance of the labeled vector in the murine brain. As a qualitative test of *in vivo* viral integrity, four Iodogen and four Bolton-Hunter mice were sacrificed 4.5 weeks after vector administration, brains were harvested and immunohistochemical staining for human TPP-1 was performed. We observed activity of gene transfer demonstrating *in vivo* function of I-124AAVrh.10CLN2 with cells in both the cortex and striatum positive for TPP-1 compared to controls (Fig. 4).

**Figure 5:**
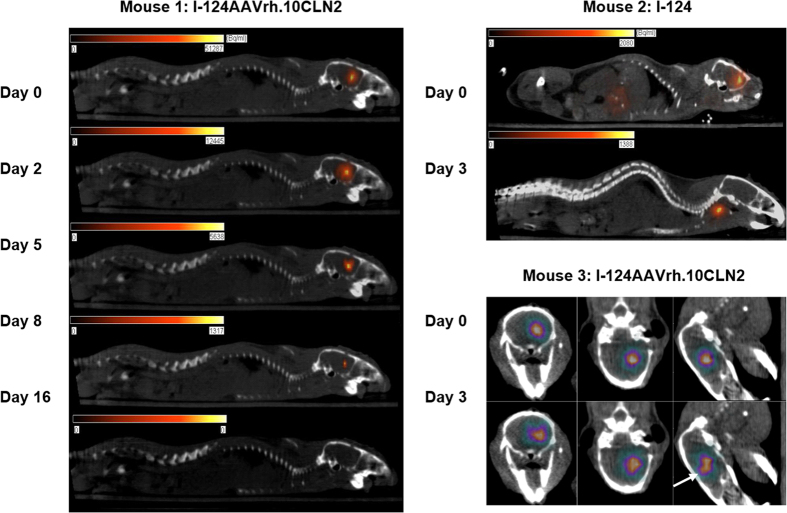
PET/CT data acquired using I-124AAVrh.10CLN2 prepared using the Iodogen method. The five images in the left column (Mouse 1) demonstrate binding of I-124 to the vector for several days after administration, while mice injected with free iodine exhibited rapid clearance from the brain and accumulation in the thyroid (Mouse 2, upper right). In the lower right panel (Mouse 3) vector has begun to spread beyond the injection site by Day 3.

PET/CT imaging clearly demonstrated the spatial and temporal distribution of vector over an eight day period, with minimal I-124 uptake in the unblocked thyroid. In contrast, free iodine was rapidly cleared from the brain within about 2 hours, and appeared in both the thyroid and liver (Fig. 5). In some cases we also observed a change in the spatial distribution of activity in the brain over time (Mouse 3, Fig. 5) suggesting that this method may be useful in monitoring vector transport in larger animal models and human studies.

**Figure 6:**
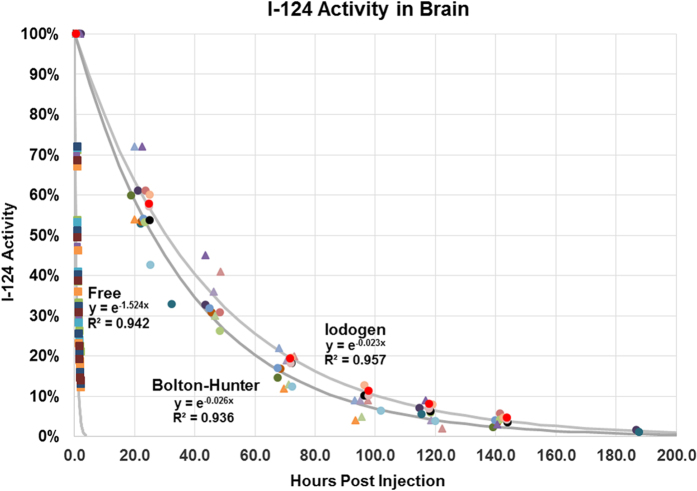
Whole brain time activity curves for I-124 using the Iodogen (N = 12, circles) and modified Bolton-Hunter (N = 6, triangles) methods, compared with free I-124 (N = 6, squares). Different colors represent individual mice.

The biological half-life T_bio_ of I-124AAVrh.10CLN2 is easily calculated from the physical half-life T_phys_ and the effective half-life T_eff_ as 1/T_bio_ = (1/T_eff_ − 1/T_phys_). For the Iodogen method we found T_bio_ = 1.83 ± 0.26 days (N = 12), and for modified Bolton-Hunter T_bio_ = 1.47 ± 0.26 days (N = 6). These values were significantly different (p = 0.02). This was in sharp contrast to T_bio_ = 0.46 ± 0.04 hours for the free iodide administrations (N = 6, Fig. 6).

## Discussion

Viral vectors have been radiolabeled for almost twenty years, using molecular imaging methods that include imaging of reporter genes^29^,^30^,^31^,^32^,^33^,^34^. Adeno-associated virus has also been labeled with quantum dots^35^. Other strategies for visualization of vector delivery have also been employed, including for example co-infusion of gadolinium loaded liposomes with AAV^36^.

A central question that arises with viral capsid labeling is how well the spatial distribution of radioactivity predicts the ultimate distribution of the protein produced by the recombinant viral gene. In the case of AAVrh.10CLN2 this question is further complicated by the fact that TPP-1 is a secreted protein. While the vector distribution is thus not expected to be a complete surrogate for the ultimate spatial distribution of gene expression, observation of the vector in the organ of interest over a one week period may still prove useful as a prognostic marker for gene delivery.

I-124 has favorable physical properties in that it is suitable for PET and the half-life is sufficiently long (T_1/2_ = 4.18 days), so that the radiochemistry is not as time critical as for example, in synthesis of compounds with C-11 (T_1/2_ = 20.4 minutes). However, the positron emitted from I-124 has a range in tissue of up to 1 cm with an average range of about 3 mm. This means that in a small animal model such as mice, there will be a spatial blurring of the distribution of radioactivity, while translation of the methods to larger animals and humans will not incur this penalty relative to the size of the brain.

The use of direct Iodogen or modified Bolton-Hunter radioiodine labeling techniques means that the entire procedure including measurement of the spatial distribution of vector *in vivo* via PET imaging has the significant advantage of requiring only a single opening of the blood-brain barrier. They are also used directly with the gene of interest, such as I-124AAVrh.10CLN2. For longer term reporting on vector distribution other methods are available. For example, the sodium iodide symporter (NIS) is a well-known and extensively studied reporter gene, and NIS cDNA can be contained within the AAVrh.10 capsid^37^,^38^,^39^. This and other reporter gene methods require a few weeks to be expressed and so can only be assessed in that timeframe.

Although iodination using Iodogen is relatively mild compared to harsher oxidizing reagents such chloramine –T, the process involves the oxidation of the iodide to iodonium cation as a reactive species. The cationic radioiodine then reacts with tyrosine residues on the capsid to form iodo-tyrosine. This process can potentially cause oxidative changes to the biomolecule. The modified Bolton-Hunter technique is a two-step procedure whereby direct exposure of an oxidant to the viral particle is avoided, and thus is considered to be a milder form of binding than the Iodogen technique. This method utilizes an iodinated intermediate and has an active ester as a reactive species. The coupling of the radioiodinated active ester to an amine function on lysine residues of the capsid facilitates radiolabeling under non-oxidizing conditions. A critical component of the radiochemistry was the ability to remove aggregates of virus from the final I-124 labeled AAVrh.10CLN2 preparation. We were able to accomplish this with both labeling methods using ion exchange chromatography followed by size exclusion methodology.

In comparing the two radioiodination methods a significant difference in the biological half-lives was observed at the level of p = 0.02, but the absolute difference in the biological half-lives was modest. This may reflect the fact that capsid uncoating occurs within a few days after administration, and as such, the results are indicative of the uncoating time *in vivo*. We observed radioactivity in the murine brain up to eight days post I-124AAVrh.10CLN2 administration. In larger animal models this may allow mapping of the spatial diffusion of vector over this time period. Since minimal activity was observed in the thyroid gland with either method, we conclude that the iodine atoms remained bound to the capsid proteins over a one week time period.

While most radioiodinated capsids appeared to retain properties sufficient for infectivity, there was a significant decrease relative to a control group for both the Iodogen and Bolton-Hunter methods (Fig. 3). Infectious entry pathways of adeno-associated viruses have been an area of intense study^40^,^41^,^42^,^43^,^44^,^45^,^46^, and for example there has been significant recent interest in modulating gene transfer of AAV2 vectors via manipulation of tyrosine residues^47^,^48^,^49^. In particular, tyrosine phosphorylation and tyrosine mutations have been studied. It is therefore not unreasonable to expect some degree of Iodogen dependent infectivity of AAVrh.10 given the above considerations. Interestingly, a similar argument can be applied to the Bolton-Hunter method that targets lysine residues on the AAV capsid. Lysine residues have also been manipulated to modulate infectivity of AAV2, and they occur with nearly the same frequency as tyrosine residues on the AAV2 capsid^50^. Conversely, it is possible to view the degree of iodine binding to tyrosine or lysine residues as potential tools to probe questions regarding infectivity, especially since binding can be quantified in terms of the average number of iodine atoms per virus particle.

It should be noted that in addition to the data presented on 24 mice, there were an additional seven mice that were studied, but for which there were problems with the injections, including needle bending (N = 2), activity on the skull indicating incomplete administration to brain (N = 2), activity in the orbit indicating incomplete administration to brain (N = 2), and one animal whose activity decreased at more than double the rate of the rest of the modified Bolton-Hunter cohort for unknown reasons. Still, we consider the surgical and injection methods to be quite robust, and we routinely performed three surgeries in succession followed by PET/CT imaging in one afternoon.

Finally, while this paper focused on the direct infusion of vector to the brain, several delivery routes to the CNS are of course possible, such as intravenous, intra-arterial, intra-cisternal, and intra-ventricular. Regarding the absolute number of virus particles injected into the brain, we achieved a radiolabeled viral titer that could be co-injected with a potentially therapeutic dose of AAVrh.10CLN2^22^. One of the long-term goals of our research is to develop non-invasive tools that will ultimately allow us to determine the best viral vector delivery route for a range of applications.

## Conclusions

Adeno-associated virus serotype rh.10 was reproducibly labeled with I-124 and its distribution in the mouse brain was observed. When compared to free I-124, which was cleared from the brain within about two hours, the labeled virus was observable up to eight days after administration. The radiolabeling approaches described herein have the potential for wide application in gene therapy trials, and in particular *in vivo* observation of radioactivity from I-124AAVrh.10 should be useful as a surrogate marker for vector distribution to the CNS in the first week after administration.

## Materials and Methods

### Animal Care

All experiments were carried out under a protocol approved by the Institutional Animal Care and Use Committee (IACUC) at Weill Cornell Medical College, and all methods conformed to IACUC guidelines.

### Virus Preparation

The AAVrh.10CLN2 vector consists of the human CLN2 cDNA expression cassette flanked by AAV2 inverted terminal repeats (ITR) and encapsidation signal, pseudotyped with the AAV non-human primate serotype rh.10 capsid protein. The expression cassette comprises the cytomegalovirus (CMV)/β-actin hybrid (CAG) promoter, the human CLN2 cDNA with an optimized Kozak sequence, and the rabbit β-globin poly (A) sequence^51^,^52^,^53^. The vector lacks genes of viral origin and was produced under Good Manufacturing Practice (GMP) conditions as previously described^51^. Briefly, HEK 293 T cells were co-transfected with the pAAV2-CAG-hCLN2 expression cassette plasmid and the AAVrh.10 helper plasmid (pPAK-MArh.10) that expresses in *trans* the AAV cap and rep and Adenovirus helper genes necessary for virus replication and production. Viral particles were purified from cell lysates 72 hours after transfection by ultracentrifugation using a discontinuous iodixanol gradient, followed by Q-HP ion-exchange chromatography. Purified AAVrh.10CLN2 virus was concentrated to 10^13^ gene copies/ml in phosphate-buffered saline (PBS) by centrifugation using a 100,000 MWCO membrane spin column (Millipore). The production of AAVrh.10CLN2 under GMP conditions met endotoxin, mycoplasma, sterility and transgene expression release criteria^51^. Transgene functionality was evaluated by TPP-1 enzymatic activity of supernatants after AAVrh.10CLN2 293-ORF6 cells infection.

### Determination of I-124AAVrh.10CLN2 vector genome titer

The I-124AAVrh.10CLN2 genome titer was determined by TaqMan real-time PCR using a CMV-chicken β-actin promoter specific primer-probe set. The CMV primers were as follows: CMV forward = GTCAATGGGTGGAGTATTTACGG, CMV reverse = AGGTCATGTACTGGGCATAATGC. Purified I-124AAVrh.10CLN2 was digested with proteinase K (Qiagen), 1 mg/ml in PBS in the presence of 0.5% sodium dodecyl sulfate and 25 mM ethylenediaminetetraacetate at 75 °C for 1 h followed by inactivation at 95 °C for 15 min. The proteinase K treated vector was used as a template for TaqMan using an AAVhCLN2 plasmid DNA of known copy number as a standard.

### Activity of I-124AAVrh.10CLN2

The activity of I-124AAVrh.10CLN2 vector was assessed *in vitro* by infecting 293ORF6 cells (10,000 genome copies/cell) in a 12-well plate. Cells were prepared in triplicate wells for each of the Iodogen and modified Bolton-Hunter methods, as well as for unlabeled vector as a control. At 72 hours post infection cells were harvested and a cell lysate was prepared by homogenization in 150 mM NaCl and 1 g/L Triton X-100 using a disposable pestle and 1.5 ml matching tube (Kimble-Kontes, Vineland, NJ) and clarified by centrifugation. Supernatants were assessed for TPP-1 activity as described previously^50^ following the procedure of Sohar *et al*.^54^. Briefly, supernatants (10 µl) were transferred to individual wells in 96-well format plates and incubated 30 min with 50 mM formate buffer pH 3.5 (20 µl) to activate TPP-1 precursor^55^. The reactions were initiated by adding 40 µl of substrate solution consisting of 250 μmol/L Ala-Ala-Phe 7-amido-4-methylcoumarin (Sigma Aldrich) in 100 mM acetate buffer (pH 4.0) containing 150 mM NaCl plus 0.1% Triton X-100. The plates were read from the bottom using 360/20 nm excitation and 460/25 nm emission filters in a CytoFluor 4000 TC plate reader (PE Biosystems). Plates were read at 10 min intervals for 40 min. The final activity of TPP-1 was calculated by measuring the change in fluorescence units (FU) per minute per milligram of protein (standardized by using a BCA protein assay kit; Pierce, Rockford, IL).

### Radiochemistry (Iodogen)

[^124^I]NaI was received as a 0.05 M NaOH solution in a volume of 50–100 μL. The pH of the radioiodine solution was adjusted to ~7.5 using pH 7.5, 250 mM tris HCl buffer containing 4 M NaCl (Stock iodination buffer) and transferred to an Iodogen coated vial. The [^124^I]NaI solution was kept in the Iodogen vial for 30 minutes at room temperature with intermittent stirring to oxidize the [^124^I]I^−^ to the reactive [^124^I]I^+^ species. The activated [^124^I]NaI solution was then carefully removed and transferred to a separate vial containing AAVrh.10CLN2. After mixing, the vial was kept at 2–5 °C on an ice bath and mixed periodically by gently stirring over 90 minutes. Following radiolabeling, the product mixture was purified using an anion exchange cartridge followed by centrifugal filtration. Purified I-124AAVrh.10CLN2 was formulated in a pH 7.4 PBS buffer. The radiolabeling yield of I-124AAVrh.10CLN2 with the Iodogen method ranged from 10–18% based on the starting [^124^I]NaI radioactivity.

### Radiochemistry (modified Bolton-Hunter)

To a dilute sodium hydroxide solution of NaI-124 (e.g. 91 μL 0.05 M), a 0.1 M acetic acid solution (e.g. 91 + 9 μL) in anhydrous acetonitrile was added and transferred to a 5 mL reaction vial and 400 μL anhydrous acetonitrile was added for azeotropic drying. The vial was placed in an oil bath at 85–90 °C and solvent was removed under a nitrogen stream. The residue was allowed to dry under a nitrogen stream for additional ten minutes. A solution (5 mg in 400 μL anhydrous acetonitrile) of a diaryliodonium salt precursor [(4-(3-N-hydroxysuccinimidylpropionyl)phenyl)(4-methoxyphenyl)iodonium triflate), (Ground Fluor Pharmaceuticals, Lincoln, NE) was added to the reaction vial and the solvent was removed under a nitrogen stream and the residue dried an additional ten minutes^56^. The residue was dissolved in 125 μL acetonitrile, 125 μL toluene was added, and the solution which was heated for thirty minutes to produce the I-124-labeled modified Bolton-Hunter reactive intermediate, I-124–2,5-dioxopyrrolidin-1-yl 3-(4-iodophenyl) propanoate (Fig. 1). After purification the reagent was recovered, reconstituted in 30–50 μL DMSO, and AAVrh.10CLN2 in native buffer was added to the vial. The mixture was incubated at ice bath temperature for 90 minutes with intermittent mixing. I-124AAVrh.10CLN2 was isolated from the reaction mixture using the same protocol as described above. The radiolabeling yield of I-124AAVrh.10CLN2 with modified Bolton-Hunter reagent ranged from 1–4.5%.

### Purification of I-124AAVrh.10CLN2

At the end of incubation, the reaction mixture was diluted with 1 mL pH 9 tris buffer and passed through a strong ion exchange cartridge (1 mL QHP cartridge, GE Healthcare). The filtrate was collected, activity measured and transferred to a 100 K MW cut-off filter (Millipore). The tube was spun at 3300 rpm for 3 minutes. The filter was washed twice with 0.5 mL aliquots of pH 7 PBS. Desired I-124AAVrh.10CLN2 was recovered from the filter in an appropriate volume of PBS. The solution was filtered through a 0.22 μm durapore membrane. The quality control of the product was established with gel electrophoresis. Radiochemically pure I-124AAVrh.10CLN2 exhibited three bands characteristic of structural proteins comprising the AAV capsid (Fig. 2).

### Labeling efficiency

The labeling efficiency of the viral capsid with I-124 was calculated as in the following examples, which applied to five mice from each of the Iodogen and Bolton-Hunter Cohorts and for which we achieved our best results. Starting with 2.6 mCi of I-124 and 1.05 × 10^12^ virus particles, the radiochemical labeling procedure was carried out using the Iodogen method. In the final product, 144 μCi were observed in a 210 microliter solution (0.69 μCi/μl), and the viral titer was 9.9 × 10^8^ particles per microliter. The total number of radioactive atoms in the product was calculated as A_o_τ where A_o_ = 144 μCi and τ is the lifetime that is related to the half-life by τ = T_1/2_/0.693. The total number of radioactive atoms bound to the AAV capsids was therefore determined to be 2.78 × 10^12^. Thus the mean number of I-124 atoms per virus particle in the preparation was 13.36. For the modified Bolton-Hunter method, starting values were 1.9 mCi and 1.05 × 10^12^ virus particles. After the labeling procedure was completed, 14.4 μCi were observed in a 25 microliter solution (0.58 μCi/μl), and the viral titer was 3.5 × 10^9^ particles per microliter. Therefore 2.78 × 10^11^ atoms were bound to AAV capsids, yielding a mean number of I-124 atoms per virus particle of 3.17. For comparison, the free iodide preparation in pH 7.4 PBS yielded 267 μCi in a 200 microliter solution (1.34 μCi/μl).

### Mouse Surgeries and Vector Administration

Adult male CD-1 mice were anesthetized with isoflurane and secured in a stereotaxic frame (David Kopf Instruments, Tujunga, CA). Two microliters of sterile formulation were unilaterally injected intraparenchymally to the left striatum (A/P +0.500 mm, M/L −2.500 mm, D/V −2.500 mm with respect to the bregma). The vector was delivered using a Hamilton Neuros syringe (Hamilton, Reno, NV) and a syringe pump (Harvard Apparatus, Holliston, MA). After the target coordinates had been reached, the needle was left in place for two minutes before starting vector delivery. The syringe pump was programmed with a two-step delivery method. Over the first 5 minutes, the flow rate ramped up from 0.01 μL/minute to 0.10 μL/minute. The flow rate was then maintained at 0.10 μL/minute until the total volume of 2.00 μL had been delivered, after which the needle was again left in place for two minutes before being raised. For the examples given above, this resulted in injected iodine activity of 1.38 and 1.16 μCi, with 1.98 × 10^9^ and 7.00 × 10^9^ genome copies per mouse respectively for the Iodogen and Bolton-Hunter methods.

### Immunohistochemistry

Assessment of TPP-1 expression mediated by the radiolabeled vector was obtained in anesthetized mice that were transcardially perfused with ice-cold PBS followed by 4% paraformaldehyde in PBS. The brains were then harvested, immersed in the same fixative overnight, equilibrated in 70% ethanol and serial 5 μm coronal paraffin sections were prepared. Immunostaining was done using a Vectastain Elite ABC kit (Vector Labs; Burlingame, CA). Briefly, slides were deparaffinized by heating at 65 °C for 20 min followed by sequential treatment with histoclear (National diagnostics, Atlanta, GA), ethanol and water. Endogenous peroxidase activity was quenched by treating the slides with 3% H_2_O_2_ solution in water. Antigen retrieval was done by treating the slides with 10 mM citrate buffer at pH 6.0. The slides were then blocked with the blocking reagent of a MOM basic immunodetection kit (Vector Labs; Burlingame, CA) and then incubated with either an anti-TPP-1 (human specific) monoclonal antibody (provided by P Lobel, Robert Wood Johnson Medical College) or an irrelevant mouse IgG (control) at 1:1000 dilution in blocking reagent. The slides were then treated with biotinylated anti-mouse secondary antibody followed by streptavidin-conjugated horse radish peroxidase. Finally, the slides were stained with DAB (diaminobenzidine) substrate. Images were obtained by brightfield microscopy with digital image acquisition using an Olympus BX50 and Microfire Camera (Olympus America Inc; Center Valley, PA).

### PET/CT

All images were acquired using Siemens Inveon PET/SPECT/CT scanner with software version 2.0. Acquisition parameters were as follows: CT: 120 projections were collected in 220 degrees of scan arc. The X-ray exposure time was 0.13 sec per projection and the X-ray tube settings were 80 kVp and 500 μA. The resulting raw data were reconstructed to a final image matrix of 480 × 480 × 667 slices at 196 μm voxel size. PET: thirty minute scans were performed and the 3D OSEM-MAP algorithm (two OSEM and 18 MAP iterations) was used to reconstruct the sinograms into a 128 × 128 × 159 matrix at 0.78 × 0.78 × 0.79 mm^3^ voxel size. CT-based attenuation compensation was performed. All mice underwent PET imaging at four or more time points.

### Image Analysis

Manual regions-of-interest of the whole brain were defined on registered Day 0 PET/CT images using the Siemens Inveon Research Workplace 4.2. Later time points PET/CT were registered with Day 0 images using a manual rigid transformation. The time activity values were calculated using the same VOIs for each animal. Exponential time activity curves were then fitted for all animals in each of three groups: free iodine, Iodogen, and modified Bolton-Hunter (Fig. 5). In fitting the exponential functions, the first point on each of the three curves was normalized to 100%. The effective half-life for each method was calculated as the time at which the exponential curve decayed to 50% of its original value.

### Biostatistics

Biological half-lives for each cohort were estimated by calculating the biological half-life for each animal using an exponential fit to the activity versus time curves, and then calculating the mean and standard deviation for the Iodogen (N = 12), modified Bolton-Hunter (N = 6), and free I-124 (N = 6) groups. P-values were estimated from a two-sample t-test assuming unequal variances using Excel. In Fig. 4, the exponential functions displayed are fitted to all data points in each of the three groups for illustrative purposes. For the infectivity assays, three measurements of TPP-1 activity were made for each of the Iodogen labeled, modified Bolton-Hunter labeled, and unlabeled vector groups, and mean values, standard deviations, and p-values were calculated as above.

## Additional Information

**How to cite this article:** Kothari, P. *et al*. Radioiodinated Capsids Facilitate *In Vivo* Non-Invasive Tracking of Adeno-Associated Gene Transfer Vectors. *Sci. Rep.* **7**, 39594; doi: 10.1038/srep39594 (2017).

**Publisher's note:** Springer Nature remains neutral with regard to jurisdictional claims in published maps and institutional affiliations.
